# Is Cannabis Abstinence Related to Subsequent Reduced Risk of Psychosis? a Nested Retrospective Case Control Hierarchical Survival Analysis

**DOI:** 10.1192/bjo.2022.192

**Published:** 2022-06-20

**Authors:** Rafael Gafoor

**Affiliations:** UCL, London, United Kingdom

## Abstract

**Aims:**

There is strong evidence from systematic reviews and meta-analyses that cannabis use is related to an elevated risk of developing a subsequent psychotic illness. It is less clear if the length of cannabis abstinence is related to the risk of developing a psychotic episode. We explore the relationship between length of cannabis abstinence and subsequent risk of a psychotic episode.

**Methods:**

We included patients aged 18–64 years who presented to psychiatric services in 11 sites across Europe and Brazil with first-episode psychosis and recruited controls representative of the local populations. We excluded all patients who reported no past history of cannabis use. We carried out hierarchical survival analyses on probability weighted data to examine the relative hazards of cessation of cannabis abstinence between cases and controls. Data on cannabis abstinence were collected retrospectively by participant recollection. We controlled for ethnicity, age, length of education and history of cigarette smoking. Proportionality assumptions were verified using Kaplan Meier plots, log-log plots and scaled Schoenfeld residuals. There was some evidence of non-proportional hazards and the assumptions of proportionality were relaxed by introducing caseness as a time varying co-variate with time specified as its natural log.

**Results:**

We included 506 cases and 482 controls with a mean age of 30.12 years (sd = 9.97). Cases had an elevated hazard ratio of cannabis use versus cannabis abstinence in controls (HR = 6.11 [SE = 1.43; p = <0.001]). There was no evidence of a difference associated with ethnicity (p = 0.198) but there were statistically significant differences at the 5% level between age groups. Ages 23–27 had a hazard ratio of 0.528 (SE = 0.064; p <0.001) versus those 18–22 years old. For ages 28–32, 33–44 and 43 to 64, the hazards ratios were 0.311, 0.110 and 0.100 (all statistically significant; p<-0.001) respectively.

**Conclusion:**

Abstinence of cannabis use is related to a reduced hazard of having a subsequent psychotic episode. The magnitude of the hazards for a subsequent psychotic illness is highest in those 18–22 years old and decreases with increasing age. Cannabis cessation maintenance therapies may have greater impact if targeting younger rather than older cannabis users who are at an elevated risk of developing a psychotic illness.

